# Co‐Culture of Mammalian Cells and Photosynthetic Microorganisms for Oxygen Supply in Engineered Tissues

**DOI:** 10.1111/cpr.70224

**Published:** 2026-05-26

**Authors:** Meng Wang, Ahmad Furqan Hala, Vera van der Niet, Sebastian T. Bok, Aylin Kara Özenler, Marcel Janssen, Maria J. Barbosa, Dirk Martens, Rene H. Wijffels, Jos Malda, Mylène de Ruijter

**Affiliations:** ^1^ Department of Orthopaedics, University Medical Center Utrecht Utrecht University Utrecht CX the Netherlands; ^2^ Regenerative Medicine Center Utrecht, Uppsalalaan 8 Utrecht CT the Netherlands; ^3^ Wageningen University Bioprocess Engineering, AlgaePARC, P.O. Box 16 Wageningen AA the Netherlands; ^4^ Department of Clinical Sciences, Faculty of Veterinary Medicine Utrecht University Utrecht CL the Netherlands

**Keywords:** cartilage, cyanobacteria, mammalian cells, microalgae, tissue engineering

## Abstract

Ensuring an adequate supply of oxygen remains a significant challenge in the development of large engineered tissue constructs in the field of tissue engineering. To address this, novel strategies have recently been introduced, including the incorporation of photosynthetic microorganisms into engineered tissues. However, to take the full advantage of this co‐culture approach, careful selection of photosynthetic microorganisms and a better understanding of their long‐term interactions with mammalian cells are required. Here, we first examined the effects of continuous 28‐day light exposure on the proliferation and biofunctionality of mammalian cells. We observed that articular cartilage‐derived chondroprogenitor cells (ACPCs) did better withstand light exposure under chondrogenic conditions than mesenchymal stromal cells (MSCs). Next, four different photosynthetic microorganisms, capable of growing at 37°C, were co‐cultured with cartilage cells. Among them, *Leptolyngbya* sp. (*Leptolyngbya*) and *Synechococcus* sp. (*Synechococcus*) did not compromise the morphology and chondrogenic capacity of mammalian cells in vitro over 28 days, whereas *Chlorella sorokiniana* (*Chlorella*) inhibited chondrogenesis. This inhibition might due to excessive oxygen release by *Chlorella* in chondrogenic culture medium, as *Leptolyngbya* and *Synechococcus* did not produce detectable oxygen under the same culture conditions. To further explore their potential for oxygen delivery to other tissue‐derived cells, we also assessed the growth rate and oxygen production of these four microorganisms in different mammalian cell culture media. We found that the composition, especially the presence of trace elements in tissue medium, critically influenced oxygen production. The tested microorganisms were able to grow and release oxygen in different mammalian cell culture media typically used for the propagation of cardiac, cartilage and liver cells, highlighting their flexible metabolic pathways across the different environments. This study emphasizes the importance of carefully selecting photosynthetic microorganisms for different tissue types, ensuring a balance between oxygen production and the specific nutritional demands of mammalian cells.

## Graphical abstract

Continuous 28‐day light exposure alters behaviours of articular cartilage progenitor cells (ACPCs) and mesenchymal stromal cells (MSCs). Co‐culture with *Leptolyngbya* and *Synechococcus* does not adversely affect the chondrogenic capacity of ACPCs over a 28‐day period, in contrast to co‐culture with *Chlorella*. The presence of tissue‐specific mammalian cell culture medium significantly influenced growth and oxygen dynamics of photosynthetic microorganisms.

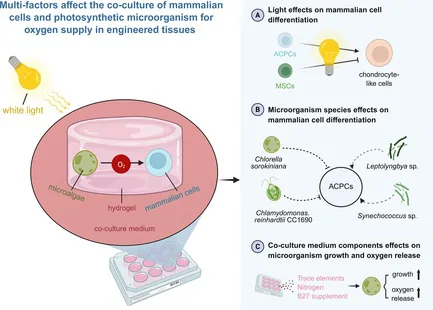

## 1 Introduction

Tissue engineering and regenerative medicine (TERM) focus on fabricating engineered tissues in both a universal and more personalized manner to better understand native tissue physiology and to facilitate in resolving donor shortages [1, 2, 3]. Biofabrication technologies now enable the precise placement of patient‐derived cells, biomaterials and bioactive molecules, offering the potential to better replicate the organization of native tissues [1]. However, the engineering of large tissue constructs, ensuring an adequate oxygen supply, still presents a significant challenge. Obviously, different tissue types do have different oxygen requirements. For example, oxygen consumption rates (OCRs) for human induced pluripotent stem cells (hiPSCs)‐derived cardiomyocytes (hiPSC‐CMs) and hepatocytes have been reported of 710 fmol cell^−1^ h^−1^ [4] and 320 fmol cell^−1^ h^−1^ [5], respectively, whereas for chondrocytes OCRs of 1–6 fmol cell^−1^ h^−1^ have been reported [6]. Moreover, fluctuations in OCR do occur during tissue development, for example, immature chondrocytes show a higher OCRs as compared to mature chondrocytes [7], highlighting the importance of ensuring a suitable oxygen supply throughout tissue development and regeneration.

Studies have focused on incorporating macro‐ and microporosity [8], vascular networks [9], as well as oxygen‐releasing systems [10] to support the oxygen requirements of engineered tissues. For example, multi‐scale vascular networks can facilitate nutrient transport [11] and cell viability can be improved through oxygen‐producing, calcium peroxide‐based particles [12]. However, introducing blood vessels into cartilage would fundamentally alter its physiological properties, since cartilage itself is avascular. Moreover, inclusion of the oxygen‐releasing calcium peroxide‐based particles could induce the production of excessive toxic H_2_O_2_. Alternatively, the incorporation of photosynthetic microorganisms, that is, green microalgae or blue–green cyanobacteria, offers an additional interesting approach [13]. These photosynthetic microorganisms can be efficiently cultured on a large scale, producing oxygen in response to exposure to light and enabling spatial patterning via 3D bioprinting techniques [14, 15]. For instance, it has been demonstrated that *Chlamydomonas reinhardtii* , a type of green microalgae, can immediately provide oxygen within a hydrogel bioink after being stimulated with light [16]. Additionally, it was shown that *C. reinhardtii* still can release oxygen after extrusion bioprinting under white light irradiation [14]. However, the influence of *C. reinhardtii* on cells can be variable, for example, sarcoma osteogenic‐2 (SaOS‐2) showed decreased cell number [14], hepatoblastoma cells (HepG2) showed im‐proved cell viability [17] when they cultured with *C. reinhardtii* . Moreover, beyond the cell‐type‐dependent effects highlighted in these studies, most in vitro experiments involving the co‐culture of photosynthetic microorganisms and mammalian cells are limited to short culture periods, typically lasting only 7 days [14, 16, 18, 19, 20, 21, 22]. Therefore, additional studies are needed to identify photosynthetic microorganisms that can sustain long‐term co‐culture with mammalian cells.

While co‐culture studies of photosynthetic microorganisms and mammalian cells have predominantly focused on species such as *C. reinhardtii* [14, 16, 17], *Chlorella sorokiniana* [18, 20, 23] and *Synechococcus elongatus* [24, 25], other species and strains remain underexplored, notably strains like *C. reinhardtii* CC 1690 (Figure S1) [26], *Leptolyngbya* sp. [27] and *Synechococcus* sp. [27]. These strains are particularly promising because they are able to produce oxygen and grow at 37°C, which is the optimal temperature for mammalian cell culture [28]. Despite these advantages, their capacity for long‐term co‐existence with the mammalian cells has not been reported yet [14, 16, 18, 19, 20, 21, 22]. Moreover, light is essential for stimulating oxygen production by photosynthetic microorganisms in co‐cultures, however, the effects of light, especially in long‐term culture periods, on the proliferation and differentiation of mammalian cells have not been fully understood. Therefore, this study aims first to evaluate the influence of continuous 28‐day light exposure on the proliferation and differentiation of different mammalian cells, including multi‐lineage potential mesenchymal stromal cells (MSCs) [29] and articular cartilage‐derived chondropro‐genitor cells (ACPCs) [30]. Next, we investigate the effects of co‐culturing mammalian cells with different microorganism species on the survival and chondrogenic capacity of engineered constructs over 28 days in vitro. Furthermore, to verify whether the four tested photosynthetic microorganisms supplied oxygen to mammalian cells in the chondrogenic condition and to explore their potential in providing oxygen to other tissue‐specific cells, both growth rate and oxygen production of these four photosynthetic microorganisms in chondrogenic medium (CM), heart medium (HM) and liver medium (LM) were investigated.

## 2 Materials and Methods

### 2.1 ACPCs and MSCs Culture

Equine ACPCs and bone marrow‐derived MSCs were isolated as previously described [30]. ACPCs were thawed and cultured in cellular expansion medium consisting of DMEM + GlutaMAX (Gibco, 31966, The Netherlands) supplemented with foetal bovine serum (FBS, 10% v/v, Biowest, S181H, France), penicillin/streptomycin (1% v/v, Gibco, The Netherlands), l‐ascorbic acid‐2‐phosphate (0.2 mM, Sigma‐Aldrich, The Netherlands), NEAA (1% v/v, Sigma‐Aldrich, The Netherlands) and basic fibroblast growth factor (1 ng mL^−1^, PeproTech, UK); ACPCs from two different equine donors were used in the light effects experiments and photosynthetic microorganism and mammalian cell co‐culture experiments, respectively. MSCs were thawed and expanded in a medium composed of alpha‐minimum essential medium (α‐MEM, Life Technologies) supplied with 10% v/v of FBS, 1% v/v of penicillin/streptomycin, 0.2 mM of l‐ascorbic acid‐2‐phosphate and 1 ng mL^−1^ of basic fibroblast growth factor. The medium was refreshed twice per week. The cells were expanded until passage 4 for further experiments.

### 2.2 Photosynthetic Microorganisms Culture

*Leptolyngbya* sp. (*Leptolyngbya*) and *Synechococcus* sp. (*Synechococcus*) were isolated from Bonaire and cultivated in a sterile artificial seawater medium enriched with nutrients (Table S1) [27]. *C. sorokiniana* (*Chlorella*) was cultivated in a modified M8 medium (Table S1) [31]. *C. reinhardtii* CC 1690 was cultivated in a modified TAP medium (Table S1) without TRIS and carbon source to promote photoautotrophic growth [32]. All media were enriched with HEPES (C_8_H_18_N_2_O_4_S, 20 mM) and 2 g L^−1^ of NaNO_3_ as the nitrogen source. Initially, the four microorganism candidates were cultivated in 250 mL flasks in triplicate under optimal conditions (37°C for all four species), using algal media (AM) as shown in Table S1. The microalgal cultures were maintained in a shaking incubator (Multitron, Infors HT, Switzerland) with 2% CO_2_ under constant agitation speed of 125 rpm, light intensity of ∼100 μmol photons m^−2^ s^−1^, with a day/night cycle of 16/8 h. Afterwards, all the algae were cultured at a temperature of 37°C since this is the suitable temperature for culturing mammalian cells. The growth rate of all the microorganisms at this temperature was assessed in a 7‐day investigation period. Next, microorganism species were cultivated in three different tissue media, that is, CM, HM and LM, for 5 days. By comparing necessary nutrient elements for growth of microalgae/cyanobacteria between AM and tissue media, we found there is an insufficient amount of nitrogen source or a non‐suitable type of nitrogen source present in the three types of tissue media for algal growth, therefore, the same experiment was repeated with addition of NaNO_3_, 2 g L^−1^ in three types of tissue media, CM, HM and LM, NaNO_3_ supplied CM, HM and LM were then marked as CN, HN and LN, respectively. Finally, for the accessibility, all the four microalgae species, *Chlorella*, *C. reinhardtii* CC 1690, *Leptolyngbya* and *Synechococcus*, which produced detectable oxygen in tissue media were cultured in suspensions in 250 mL of sterile Erlen‐meyer flasks at room temperature with waterproof clear‐white LED continuous light (LedstripKoning, the Netherlands) at around 40 μmol photons m^−2^ s^−1^.

### 2.3 Co‐Culture of Mammalian Cells With Distinct Photosynthetic Microorganisms Under Light

For the co‐culture system, microorganisms and ACPCs were embedded into gelatin methacryloyl (GelMA) hydrogels. GelMA (degree of functionalization = 80%) was synthesized as previously described [33, 34]. Irgacure2959 (I2959, Ciba Inc., Basel, CH) was dissolved in PBS to obtain a 0.1% (w/v) solution and placed in a 37°C incubator on a tube rotator until fully dissolved. The solution was filtered through a 0.22 μm filter and added to freeze‐dried GelMA to prepare a 10% (w/v) solution, which was further rotated until fully dissolved. The final cell concentration of photosynthetic microorganisms in all casting experiments was 10^7^ cells mL^−1^. Microalgae/cyanobacteria cells capable of releasing oxygen into the tissue medium were first added to 1.6 mL of GelMA solution to reach a final concentration of 1.0 × 10^7^ photosynthetic microorganisms mL^−1^ and then mixed with mammalian cells (MSCs or ACPCs) to reach a final concentration of 2.0 × 10^7^ cells mL.

A total of 1 mL of the cell suspension was injected into 10‐piece Teflon moulds with an individual diameter of 6 mm and a height of 2 mm. The Teflon moulds were placed under a UV lamp (UVP CL‐1000 Ultraviolet Crosslinker, 120,000 μJ cm^−2^) for 15 min to crosslink. Afterwards, all constructs containing photosynthetic microorganisms and mammalian cells were collected and co‐cultured in CM supplemented with 1.0 g L^−1^ NaNO_3_ (Figure S2), with or without LED continuous light (HWCS600‐03M, LedstripKoning, Netherlands) at about 40 μmol photons m^−2^ s^−1^ of light intensity. Light intensity was confirmed using a light meter (LI‐250A, LI‐COR).

The CM consisted of high‐glucose DMEM (Gibco, 31966, Life Technologies), 1% v/v ITS + Pre‐mix Universal culture supplement (Corning, USA), 0.2 mM l‐ascorbic acid‐2‐phosphate (Sigma Aldrich, The Netherlands), 0.1 μM dexamethasone (Sigma Aldrich, USA), 100 U mL^−1^ penicillin, 100 μg mL^−1^ streptomycin (Gibco, Life Technologies) and 10 ng mL^−1^ recombinant human transforming growth factor‐*β*1 (TGF‐*β*1, Peprotech, UK). For ACPC‐containing samples, 1% v/v HEPES (1 M, Gibco, Thermo Fisher Scientific, USA) was also added to the CM.

Samples were cultured for 4 weeks and harvested at Days 1, 14 and 28 for subsequent analysis. Medium was re‐freshed twice per week. All cell cultures were performed under sterile and normoxic conditions at 37°C and 5% CO_2_.

### 2.4 Viability of Photosynthetic Microorganisms and Mammalian Cells

After casting or 3D bioprinting, algae/cyanobacteria were incubated in the ACPCs differentiation medium at 37°C under illumination by an LED light stripe (light intensity 37.8 μmol photons m^−2^ s^−1^). The viability and growth of microalgae were evaluated by measuring the OD at 750 nm at different time points. Live/Dead assay was performed for examination of live and dead mammalian cells, where Calcein AM (2 μM, Invitrogen, Thermo Fisher Scientific, USA) stains the cytoplasm of live cells while ethidium homodimer‐1 (1 μM, Invitrogen, Thermo Fisher Scientific, USA) stains the nucleus of dead cells. The excitation/emission of Calcein AM and Ethidium homodimer‐1 is 499/568 and 578/700 nm, respectively. DAPI (3.375 μM, Sigma‐Aldrich, USA) was also used to stain the nucleus and excitation/emission is 410/499 nm. The viable microorganisms can be traced by the autofluorescence of chlorophyll under red to infrared wavelengths, which was detected with excitation/emission of 701 nm/800 nm. The samples were analysed using Confocal microscopy (Leica Microsystems, Germany).

### 2.5 Measurement of Chlorophyll *a*

Chlorophyll *a*, one of the very important pigments for microalgae/cyanobacteria to do photosynthesis, thus chlorophyll *a* from both mammalian cells co‐cultivations and sole constructs were extracted at Days 1, 14 and 28 using an ethanol extraction method [35] for confirming the survival and growth of photosynthetic microorganisms. One millilitres of 100% ethanol was added to each of the samples and homogenized to extract green pigments at room temperature. It was then centrifuged at 10,000 rpm for 2 min and absorption was measured at 750, 664, 647 and 630 nm with a CLARIOstar Plus microplate reader (BMG LABTECH, Germany). The content of chlorophyll *a* was calculated by the following Equation (1):

(1) $$\mathrm{Chl}\;a\;\left(\mathrm{mg}\;L^{-1}\right)=\left(11.85\times A_{664}\right)–\left(1.54\times A_{647}\right)–\left(0.08\times A_{630}\right)$$

Particularly, releasing all the encapsulated algae from GelMA hydrogel before the extraction of chlorophyll *a* is necessary. Therefore, 3D constructs were first hydrolyzed under 37°C for 2 h; the enzyme was 1600 U mL^−1^ of Collagenase Type II (Col II) (Worthington-Biochem.com) in PBS.

### 2.6 Biochemical Assays

Metabolic activity assays were performed after 1, 14 and 28 days of culture on the same samples (*n* = 5) using a resazurin assay (resazurin sodium salt, Alfa Aesar, Germany). A working solution was prepared in chondrogenic differentiation medium without TGF containing 44.11 μM resazurin sodium salt. Briefly, samples were incubated, protected from light, for 4 h at 37°C. Fluorescence was measured in duplo with excitation at 544 nm and emission at 620 nm. The dye 1,9‐dimethylmethylene is a thiazine chromotrope agent that presents a change in the absorption spectrum due to the induction of metachromasia when bound to sulphated GAGs, enabling rapid detection of GAGs in solution [36]. Therefore, the dimethyl methylene blue (DMMB) assay was used to quantify the formation of GAGs. To quantify GAGs production during the culture period, samples were taken after 1 and 28 days of culture and subsequently freeze‐dried (*n* = 3). Samples for the light effect on mammalian cells were directly digested using a papain buffer consisting of 0.2 M NaH_2_PO_4_ and 0.1 M EDTA 2 H_2_O at pH 6.0, mixed with 7.75 U mL^−1^ papain solution and 1.57 mg mL^−1^ cysteine HCl and incubated overnight at 60°C.

Samples from the photosynthetic microorganism mammalian cell co‐culture experiments were digested in 0.2 mL of Col II (1600 U mL^−1^) in PBS solvent at 37°C for about 2 h to allow complete dissolution. Next, the collagenase‐digested samples were spun down at 8000 g for 15 min. The supernatant was removed without disturbing the pellet, after which 0.2 mL of 0.5 mg mL^−1^ proteinase K solution (in PBS) was added and the samples were digested overnight at 60°C.

Next, the digested samples were assayed for DNA and GAGs content using the Picogreen assay kit (Thermo Fisher Scientific) and DMMB assay (Sigma), respectively. Briefly, DMMB solution was prepared in‐house with a pH of 3.0. Chondroitin sulphate C was used to prepare a standard curve with concentrations from 0 to 10 μg mL^−1^. Excitation was measured in duplo at 525 and 595 nm wavelengths, dividing the 525 nm measurement by the 595 nm measurement before subtracting the blank and performing subsequent analysis. For Picogreen, Quant‐iT Picogreen kit stock of *λ*DNA (100 μg mL^−1^) in 1× TE buffer was used to create dilutions for standard curve with concentrations from a range 0 to 2000 ng mL^−1^. Excitation was measured at 485 and 520 nm emission. GAGs production for co‐cultured samples was normalized by the dry weight of the samples.

### 2.7 Histology and Immunohistochemistry

After 1, 14 and 28 days of culture, samples were fixed in formalin for 24 h and then transferred to a tissue processor (Leica ASP300S, Germany) for general dehydration. Samples were subsequently embedded in paraffin. Following paraffin embedding, samples were cut at 5 μm thickness and stained with safranin‐O for glycosaminoglycan (GAG) visualization, fast green for cytoplasm and collagen and Weigert's haematoxylin for cell nuclei. HiF‐1*α* transcription factor is a classic important hypoxia mediator in chondrocytes [37], HBB is currently discovered in the cytoplasm of chondrocytes and is considered an oxygen reservoir for chondrocytes [38], both HiF‐1*α* and HBB can be upregulated upon hypoxia. Thus, HiF‐1*α* and HBB were also stained to help us detect the influence of oxygen on the mammalian cells. Here the primary antibody polyclonal anti‐HiF‐1*α* (PA1‐16627, Thermo Fisher), HBB‐antibody‐polyclonal (PA5‐119182, Thermo Fisher) and Col II (II‐II6B3, DHSB) were used in 1:200, 1:100 and 1:100 dilution, respectively. Histology images were made of mounted sections in 3× random locations using a bright field microscope (Olympus BX51, The Netherlands).

### 2.8 Measurement of Cell Number and Growth Rate of Microorganisms

The optical density at 750 nm (OD_750_) was chosen to assess growth parameters and measurements of growth kinetics [39]. The growth rate of the microalgae was calculated by plotting OD_750_ versus the days of cultivation on a logarithmic scale. The growth rate (*μ*) is the slope of the plot and can be expressed by Equation (2). Here, *N*_*tx*_ and *N*_*t*0_ represent the OD_750_ values at the day of measurement (*t*_*x*_) and the initial day zero (*t*_0_), respectively.

(2) $$\;\mu \left({\mathrm{day}}^{-1}\right)=\frac{\ln\left(N_{\mathrm{tx}}/N_{t0}\right)}{t_{\mathrm{tx}}-t_{t0}}$$

OD750 was measured using a UV/Vis spectrophotometer (DR6000, Hach, Manchester, UK) or Multiplate Reader (Infinite 200 Pro, Tecan, Switzerland). The microalgal or cyanobacterial samples were put in a 1 mL cuvette or 96‐well plate and diluted with seawater/freshwater (depending on the species measured) when the measurement was outside the range of 0.1–0.3 (cuvette) or over 0.1 (well‐plate). To calculate the cell number and biomass of the microorganisms, before related experiments, the samples were measured using a plate reader spectrophotometer (Infinite 200 Pro, Tecan, Switzerland) and a correlation was made between the UV/Vis and the plate reader spectrophotometer. Each measurement was done in duplicate, and the average value was then converted to OD_750_ from the UV/Vis spectrophotometer according to the related formulas (Tables S2 and S3).

### 2.9 Specific Oxygen Production Rate (SOPR) Measurement

The SOPR of the microalgae or cyanobacteria, which showed growth in the tissue media (both the supplied tissue media and the nitrogen‐enriched media) was measured using a biological oxygen monitor (BOM) equipment (Oxytherm + Photosynthesis, Hansatech Instruments, UK). As a comparison, the oxygen production rate from the microalgae or cyanobacteria growing in algal medium was also measured. The oxygen electrode for the chamber was first prepared by putting five drops of 2.35 M KCl solution onto the electrode and layering it with around 2 × 2 cm rolling paper (Rizla, UK) and the same size of S4 PTFE Membrane (Hansatech Instruments, UK). The layers on the electrode were then secured with rubber rings and connected to the BOM chamber. The light inside the chamber was automatically calibrated with QTP1 probe sensor (Hansatech Instruments, UK). For every different growing medium used during the measurement, a calibration for oxygen concentration was done for the oxygen‐saturated condition and zero oxygen condition in the chamber. The oxygen‐saturated condition was reached by sparging the chamber with air until the reading reached a steady state (changes of less than 1 nA min^−1^). Then, the chamber was sparged with nitrogen and a pinch of sodium hydrosulfite (Na_2_S_2_O_4_, technical grade, Sigma‐Aldrich, USA) was also added to reach zero oxygen conditions. When the electrode reading once again reached a steady state, the calibration was saved.

During this experiment, the microorganisms were cultivated for 5 days in the tissue media (plain tissue media or tissue media supplemented with additional nitrogen sources). Around 2.5 mL culture in 12‐well plates was prepared with an initial biomass concentration of 0.1 g L^−1^. The well plates were secured with Parafilm to prevent evaporation. For the preparation of the sample, 1 mL of culture from the experiments was taken and the OD_750_ was measured. The biomass dry weight was then determined using the previously found correlation. A similar growing medium was also prepared by putting it in the shaking incubators (Multitron, Infors HT, Switzerland) with 2% CO_2_ under a constant agitation speed of 125 rpm. The medium preparation was conducted to prevent bicarbonate limitation during the BOM measurement. The biomass sample was then diluted with the medium to reach a biomass concentration of around 0.1 g L^−1^ in the BOM chamber. The chamber was then sparged with nitrogen gas to remove all the initial dissolved oxygen. Once the initial OD was 0, a set of photon flux density (PFD) plans was then run in the chamber with an agitation rate of 50 rpm and a temperature of 37°C. The PFD plan for the oxygen production rate measurement can be seen in Table S4. The oxygen concentration from the BOM was modelled according to the model of Webb [40] to find the oxygen production rate.

### 2.10 Statistical Analyses

Data is presented as mean ± standard deviation, unless otherwise specified. Significant differences were evaluated statistically by GraphPad Prism10 (California, US) using Two‐way ANOVA with Tukey's multiple comparisons test, unless otherwise specified. Differences were considered significant when *p* ≤ 0.05. Significance is indicated on graphs as follows: *p ≤ 0.05, **p ≤ 0.01, ***p ≤ 0.001, ****p ≤ 0.0001, ns: no significant difference.

## 3 Results and Discussion

### 3.1 Influence of Continuous Light Exposure on Mammalian Cells

White light is commonly used to cultivate photosynthetic microorganisms [41, 42] and has also been used to adjust oxygen supply to mammalian cells in photosynthesis‐based co‐cultures [16, 22, 24]. However, the effects of continuous white light exposure for more than 14 days on biofunctionality of mammalian cells remain unclear [21, 22, 43]. Present study evaluated 28 days of continuous white light illumination effects on the chondrogenic differentiation capacity of 3D‐cultured ACPCs and MSCs.

After 28 days of culture under standard (dark group) and light exposure (light group) conditions, ACPCs differentiated, as indicated by the positive staining for GAGs (Figure 1A). From Days 1 to 28, DNA content of ACPCs was comparable for the two groups throughout the culture period (Figure 1B), suggesting that light had negligible effects on proliferation of ACPCs. Production of GAGs in the dark group increased significantly over the culture period (Figure 1C), while the light‐exposed group maintained relatively stable levels. By Day 28, production in the light group was significantly lower than in the dark group (*p* < 0.001, Figure 1D). This suggests that ACPCs GAGs production may be hampered by light, specifically after prolonged culture.

**FIGURE 1:**
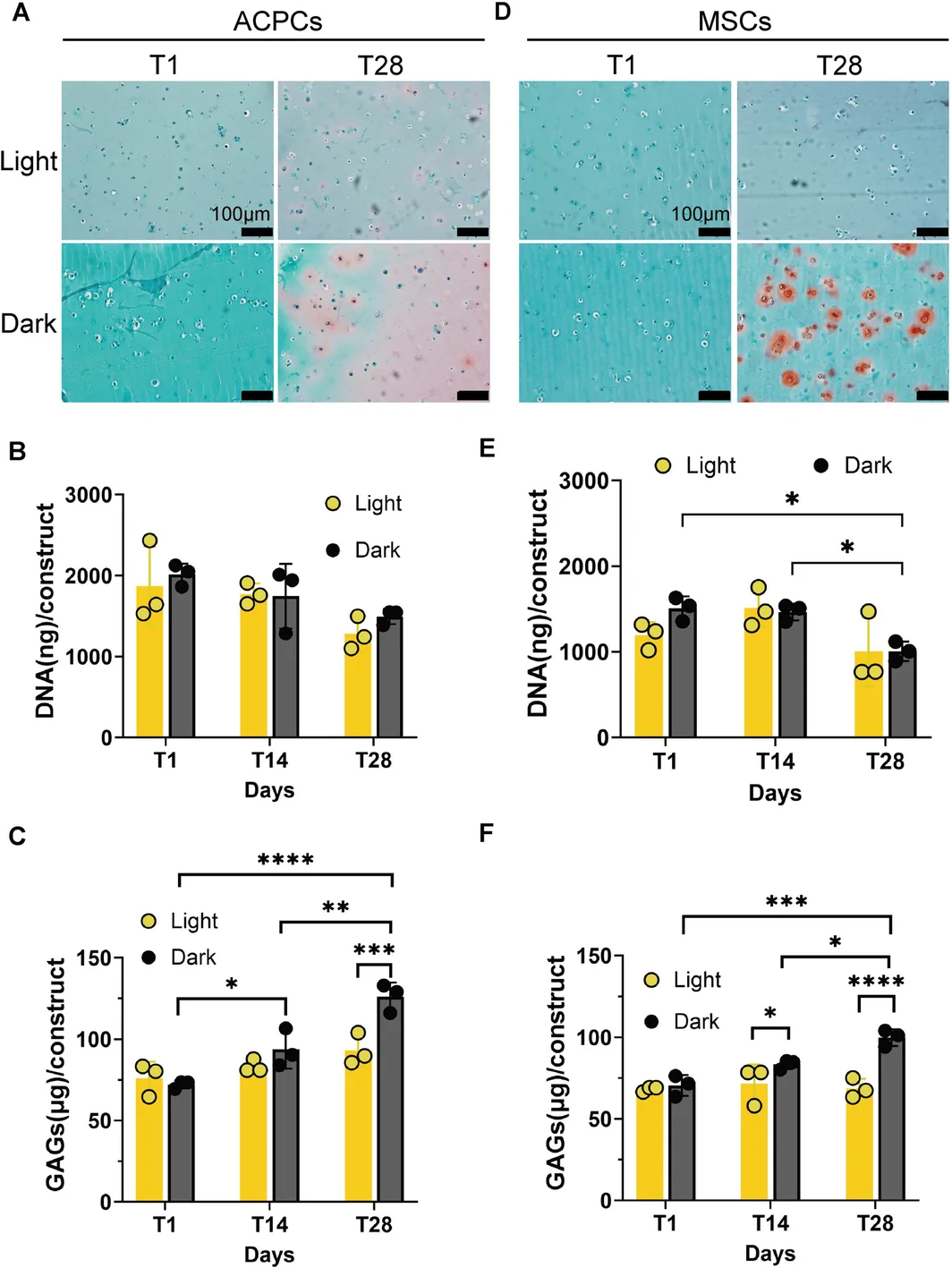
Effect of continuous light exposure on the proliferation and chondrogenic differentiation of ACPCs and MSCs over a 28‐day culture period. (A) Safranin‐O staining showing glycosaminoglycans (GAGs) formed by ACPCs within constructs (*n* = 2). Scale bars: 100 μm. (B, C) Biochemical analysis of ACPCs‐containing constructs: (B) DNA content per construct (*n* = 3), and (C) GAGs content per construct (*n* = 3). Two‐way ANOVA with Tukey's multiple comparisons test. (D) Safranin‐O staining of GAGs formed by MSCs within constructs (*n* = 2). Scale bars: 100 μm. Biochemical analysis of MSC‐containing constructs: (E) DNA content per construct (*n* = 3, two‐way ANOVA with uncorrected Fisher's LSD) and (F) GAGs content per construct (*n* = 3, two‐way ANOVA with Tukey's multiple comparisons test). *p ≤ 0.05, **p ≤ 0.01, ***p ≤ 0.001, ****p ≤ 0.0001.

Regarding MSCs, after 28 days, GAG production in the dark showed signs of chondrogenic differentiation, as indicated by positive staining for GAGs, however, no staining of GAGs was observed in the light‐exposed group (Figure 1D). Similar to ACPCs, light had limited effects on the proliferation of MSCs but a significant impact on GAGs formation, as indicated by the comparable DNA content (Figure 1E) and significant difference of GAGs content (*p* < 0.0001 on Day 28) between light and dark groups (Figure 1F).

Overall, these findings suggest that light has a significant effect on GAGs synthesis in mammalian cells during chondrogenic differentiation. These observed suppressive effects of white light on mammalian cells might stem from several factors and are likely cell and tissue‐type specific. These factors likely include the blue and green light portion in white light [21, 44], which have been reported to induce cytotoxicity via increased ROS generation, impaired ATP production, reduced proliferation in stem cells [45, 46]. Additionally, continuous illumination, as it has been reported to adversely affect cellular behaviour compared to dark conditions [21] and pulsed illumination should be investigated. In future studies, the use of red light/near‐infrared [21, 25, 46], pulsed illumination, for example, 12 h light/12 h dark [22] and lower light intensity [47] could potentially alleviate the harmful effects of white light on cells while sustaining the photosynthetic function of photosynthetic microorganisms. Yet, it is expected that all these factors are cell‐and tissue‐type‐specific.

In short, we found continuous 28‐day white light irradiation can significantly impair the chondrogenic functionality of ACPCs and MSCs; however, ACPCs outperformed MSCs and thus ACPCs were used for further studies.

### 3.2 Co‐Culture of Photosynthetic Microorganisms and Mammalian Cells

Four photosynthetic microorganisms were co‐cultured with ACPCs in GelMA hydrogels to investigate the effects of microorganism species on the survival of engineered tissue constructs. These four microorganisms including *Chlorella*, *C. reinhardtii* CC 1690, *Leptolyngbya* and *Synechococcus*. The morphology of them shown in Figure 2A.

**FIGURE 2:**
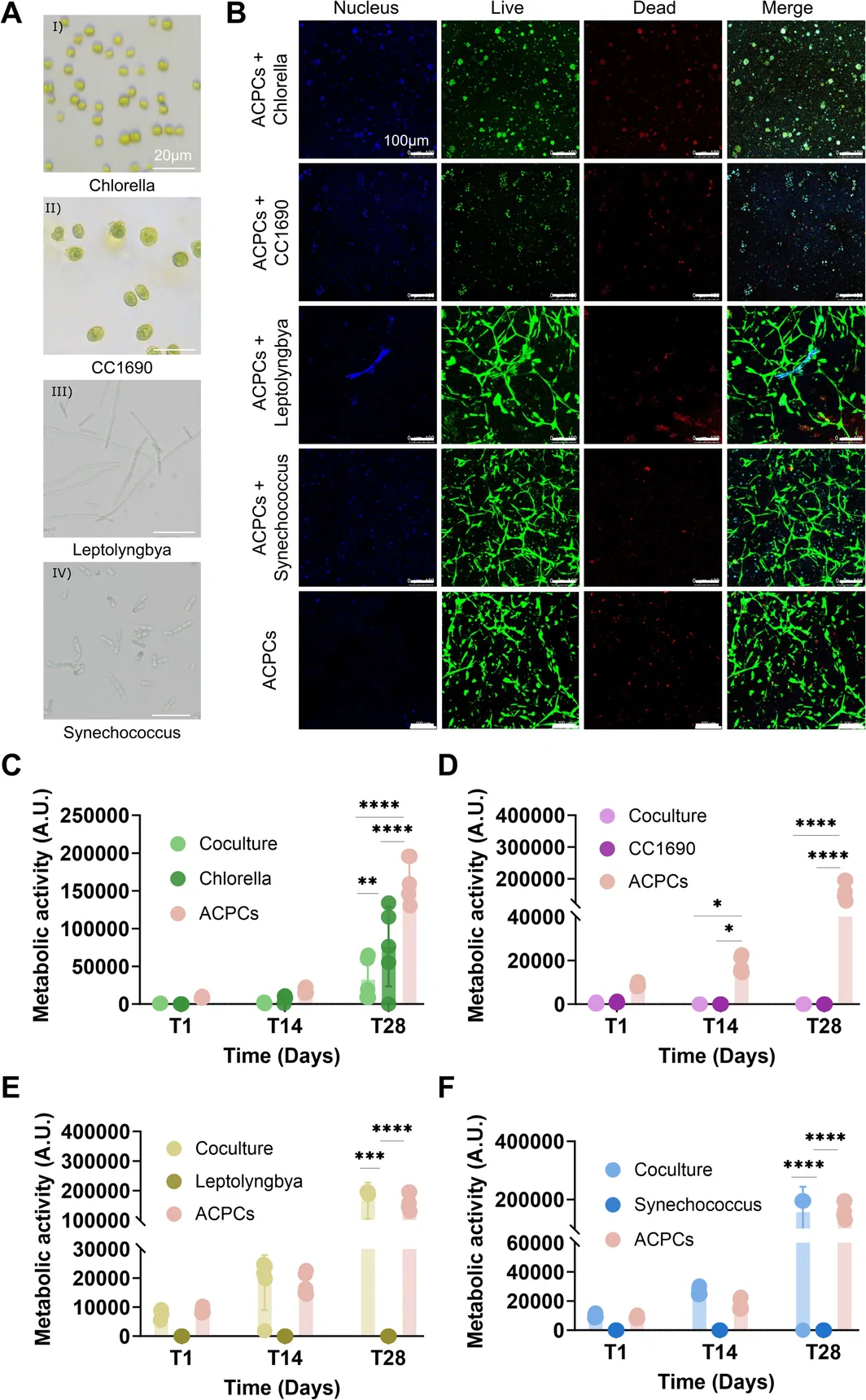
Co‐culture of photosynthetic microorganisms and ACPCs in chondrogenic medium during 28‐day continuous light exposure. (A) Microscopy images of four photosynthetic microorganisms that can grow under 37°C. Scale bars: 20 μm. (B) Live/dead staining of various photosynthetic microorganisms incorporated ACPCs constructs on Day 28 (*n* = 3). Scale bars: 100 Μm. Metabolic activity of microorganisms incorporated ACPCs constructs based on *Chlorella* (C), *Chlamydomonas reinhardtii* CC 1690 (D), *Leptolyngbya* (E) and *Synechococcus* (F) (*n* = 5). Two‐way ANOVA with Tukey's multiple‐comparisons test was applied to (C–F). *p ≤ 0.05, **p ≤ 0.01, ***p ≤ 0.001, ****p ≤ 0.0001.

Abundant nitrogen sources are critical for the growth and photosynthesis of photosynthetic microorganisms [48, 49, 50, 51]. We found nitrogen concentration in basic CM is about five orders lower than in AM used for culturing photosynthetic microorganisms (Table S5). Therefore, additional nitrate supplementation is necessary for the co‐culture of photosynthetic microorganisms and mammalian cells. Before supplementation, we evaluated the effect of different nitrate concentrations on ACPCs' metabolic activity. Our results showed that all tested nitrate concentrations, ranging from 0.25 to 2 g L^−1^, increased metabolic activity from Days 1 to 14 compared to the control (Figure S2), likely due to enhanced mitochondrial efficiency induced by nitrate treatment [52, 53]. Concentration of 1 g L^−1^ of nitrate was then selected for the CM used for co‐culture experiments, as it not only exhibited the greatest enhancement of ACPCs metabolic activity over 14 days (Figure S2), but also within ranges commonly used for microalgal and cyanobacterial cultures without reported adverse effects on growth or oxygen evolution [54, 55, 56, 57].

After 28 days, co‐culture of ACPCs with *Chlorella* and *C. reinhardtii* CC 1690 (Figure 2B) showed distinct phenotypes compared to ACPCs‐only constructs. Moreover, these two types of co‐cultured constructs exhibited significantly lower metabolic activity (Figure 2C,D) compared to constructs containing only photosynthetic microorganisms or ACPCs alone over the 28 days. Notably, the metabolic activity of *Chlorella*‐based co‐cultured constructs was five times lower than that of the ACPCs‐only group after 28 days. In contrast, no detectable metabolic activity was observed in *C. reinhardtii* CC 1690‐based constructs, whether containing mammalian cells or not, after 14 days of culture. Moreover, these constructs containing *C. reinhardtii* CC 1690 showed a continuous decrease in chlorophyll *a* content in nitrate‐supplied CM over 28 days (Figure S3B,F), indicating that at least a part of the *C. reinhardtii* CC 1690 population may have died within the constructs. This observation aligns with previous findings showing that penicillin/streptomycin‐supplemented medium negatively affects the growth of *C. reinhardtii* , where *C. reinhardtii*‐laden constructs initially appeared light green on Day 7 but turned completely white by Day 14 [17]. Moreover, antibiotic stress and associated ROS [58] in *C. reinhardtii* may not only hamper its growth and photosynthetic activity [59], but also could affect the surrounding cells through ROS wave signals [60]. This may further explain the altered phenotype, reduced viability and suppressed metabolic activity of ACPCs observed when co‐cultured with *C. reinhardtii* CC 1690. Therefore, application of *C. reinhardtii* CC 1690 was not evaluated further for the chondrogenic functionality experiment.

Although *Chlorella* may also suffer from antibiotic stress, it shows significantly higher tolerance compared to *C. reinhardtii* [61], which is consistent with the observations in the present study. For constructs containing *Chlorella*, there was a significant increase in chlorophyll *a* content from Days 1 to 14 (*p* < 0.001), followed by a significant decrease from Days 14 to 28 (*p* < 0.01), as indicated by Figure S3A. Moreover, the chlorophyll *a* content of *Chlorella* in co‐cultured constructs was significantly higher than in those with *Chlorella* alone (*p* < 0.0001, Figure S3A and *p* < 0.01, Figure S3E), indicating that *Chlorella* can survive and grow within the co‐cultured constructs. However, the differences in ACPCs phenotype and the lower metabolic activity after 28 days in the *Chlorella*‐based co‐cultured construct, as compared to the ACPCs‐only constructs, suggest the need to optimize the co‐culture system. This optimization is essential for achieving better integration of *Chlorella* and mammalian cells.

Additionally, after 28 days, co‐culture of ACPCs with *Leptolyngbya* and *Synechococcus* preserved their phenotype (Figure 2A) and showed similar metabolic activity compared to the culture of ACPCs alone (Figure 2E,F), it implies that the influence of *Leptolyngbya* and *Synechococcus* on ACPCs is quite limited. Notably, neither *Leptolyngbya* nor *Synechococcus* alone exhibited detectable metabolic activity from Days 1 to 28. The absence of measurable metabolic activity in both microorganisms is likely due to their small algal‐cell size (Figure 2A) and the low number of cells presented in the construct (Figure S4).

Furthermore, the extraction of chlorophyll *a* from the *Synechococcus*‐based construct over 28 days (Figure S3D,H) suggested that *Synechococcus* survived within the construct. When co‐cultured with mammalian cells, the chlorophyll *a* content in the *Synechococcus* was comparable to that in the microorganism‐alone constructs (Figure S3D,H). These findings indicate that *Synechococcus* are likely coexist with chondrogenic mammalian cells and that the engineered co‐culture constructs can survive and grow throughout the 28‐day culture period.

### 3.3 Functionality of Co‐Cultured Photosynthetic Microorganisms and ACPCs Constructs

Chondrogenic functionality of co‐cultured microorganisms and ACPCs was evaluated by histology staining and biochemical tests. After 28 days, the ACPCs‐only construct exhibited positive GAGs staining (Figure 3A). In contrast, the co‐cultured construct containing ACPCs and *Chlorella* did not show any production of GAGs, as shown through safranin‐O staining (Figure 3A). The positive Safranin‐O staining observed around the *Chlorella* colonies in the co‐culture group were likely due to the presence of lignin [62] and sulphated polysaccharides [63] in the algal cell wall, a phenomenon also noted in the *Chlorella*‐only group (Figure 3A). Conversely, both *Leptolyngbya*‐ and *Synechococcus*‐ based co‐cultured constructs displayed similar positive staining and a general distribution of cartilage‐like GAGs as the ACPCs‐only group (Figure 3A). No similar signs were observed in the *Leptolyngbya*‐ and *Synechococcus*‐only constructs.

**FIGURE 3:**
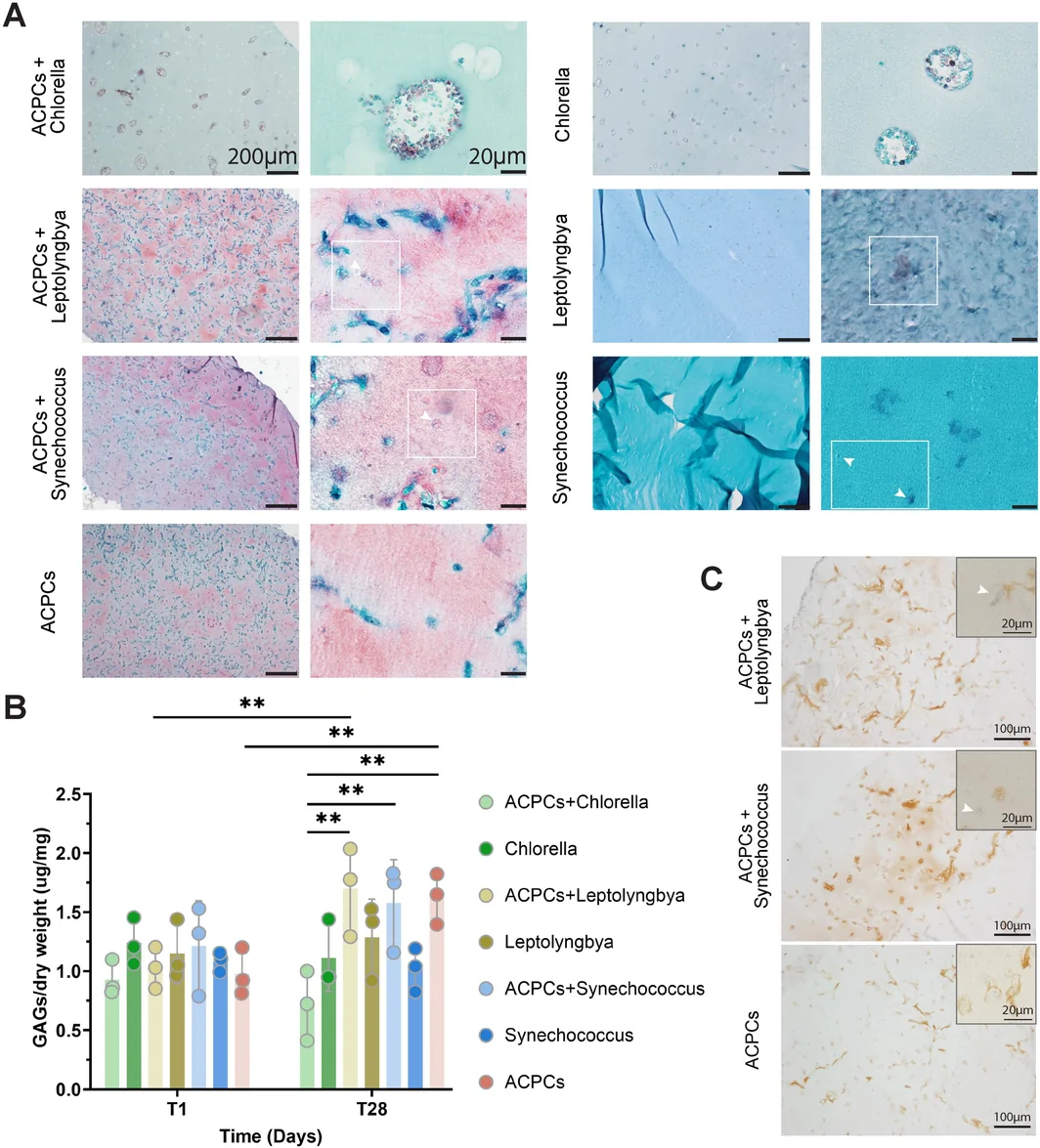
Chondrogenic differentiation of ACPCs when co‐cultured with photosynthetic microorganisms under continuous 28‐day light exposure. (A) Safranin‐O—Fast Green staining of GAGs within co‐cultured constructs (*n* = 3). Scale bars: 200, 20 μm. The white arrows indicate *Leptolyngbya* or *Synechococcus* inside of constructs. (B) GAGs quantification of co‐cultured constructs (*n* = 3) (C) Collagen type II staining of cultured constructs (*n* = 3). Scale bars: 100 μm for overview images, 20 μm for zoom in images (up right). Two‐way ANOVA with Tukey's multiple comparisons test. **p ≤ 0.01.

Quantification of GAGs showed similar levels across all the construct groups on Day 1 (Figure 3B). After 28 days, the co‐culture of ACPCs with *Chlorella* showed GAGs levels comparable to the *Chlorella*‐only group, but significantly lower than the ACPCs alone group (*p* < 0.01, Figure 3B). In contrast, both *Leptolyngbya*‐ and *Synechococcus*‐based co‐culture groups displayed GAGs content similar to that of the ACPCs alone group (Figure 3B). Notably, all microorganism‐only groups displayed GAGs levels comparable to the ACPCs‐only group on Day 28 (Figure 3B), however, no cartilage‐like GAGs were observed in the Safranin‐O staining (Figure 3A). Amounts of GAGs quantified in Figure 3B were likely influenced by the sulphated polysaccharides found in the green microalgae or cyanobacteria [63, 64, 65, 66, 67]. These results indicate that both *Leptolyngbya* and *Synechococcus* have limited effects on the chondrogenesis of ACPCs during the 28‐day co‐culture period, whereas *Chlorella* significantly hampered the chondrogenesis of ACPCs under the current co‐culture conditions. This is further supported by Col II staining, where both *Leptolyngbya*‐ and *Synechococcus*‐based co‐culture constructs showed positive signs similar to ACPCs‐only group (Figure 3C). However, the *Chlorella*‐based co‐culture construct and all the microorganism‐only constructs demonstrated negative results in Col II staining (Figure S5).

Chondrogenesis shown in *Leptolyngbya*‐ and *Synechococcus*‐based co‐culture constructs are probably due to the limited effects of oxygen released and supplied by these microorganisms on ACPCs under the current co‐culture conditions. This is demonstrated by the presence of both HIF‐1*α* and HBB in Figure S6. In contrast, the inhibited chondrogenesis observed in *Chlorella*‐based co‐culture constructs might result from the excessive oxygen supplied by *Chlorella* during the 28‐day cultivation period. As shown in Figure S6, there were no indications of HIF‐1*α* or HBB in the *Chlorella*‐based co‐cultured construct after 28 days, although these factors were present on Day 1. Another possible explanation for the differences in chondrogenesis could be the limited availability of nutrients, for example, glucose, for ACPCs in the *Chlorella* co‐cultured constructs. Glucose is essential for synthesizing extracellular matrix GAGs in cartilage [68]. *Chlorella* exhibits mixotrophic growth [65], where it simultaneously consumes glucose for heterotrophy and performs photoautotrophy under light conditions. This mixotrophic behaviour may explain the significant increase in chlorophyll *a* content (*p* < 0.001, from Days 1 to 14) followed by a decrease (*p* < 0.01, from Day 14 to 28), as observed in Figure S3. However, further studies are needed, as the presence of glucose may also influence oxygen production by *Chlorella* [18].

### 3.4 Growth and Net Oxygen Production of Photosynthetic Microorganisms in Tissue‐Specific Media

To determine if the selected photosynthetic microorganisms can release and supply oxygen to ACPCs under chondrogenic conditions and to examine their potential to provide oxygen to other tissue‐derived cells, we investigated the growth rate and oxygen production of various strains in different media: CM and CN, HM and HN, as well as LM and LN. Although *C. reinhardtii* CC 1690 was excluded in Section 3.3 due to significant negative effects on the survival and metabolic activity of ACPCs, another strain of *C. reinhardtii* has been reported to supply oxygen to liver cells over a 7‐day study [17]. Therefore, we did explore whether *C. reinhardtii* CC 1690 can supply oxygen to distinct tissue‐specific cells.

As shown in Figure 4A, all four photosynthetic microorganisms demonstrated the capacity to release oxygen in their respective culture medium (AM) at 37°C. Moreover, the net SOPR increased with higher light intensity. For *Chlorella*, *Leptolyngbya* and *Synechococcus*, the maximum net SOPR were reached under 1500 μmol photons m^−2^ s^−1^ of light intensity and the maximum net SOPR were 1.493 ± 0.086, 1.286 ± 0.085 and 1.117 ± 0.012 μmol O_2_ g^−1^ s^−1^, respectively. In contrast, only 0.083 ± 0.003 μmol O_2_ g^−1^ s^−1^ of maximum net SOPR was achieved for *C. reinhardtii* CC 1690.

**FIGURE 4:**
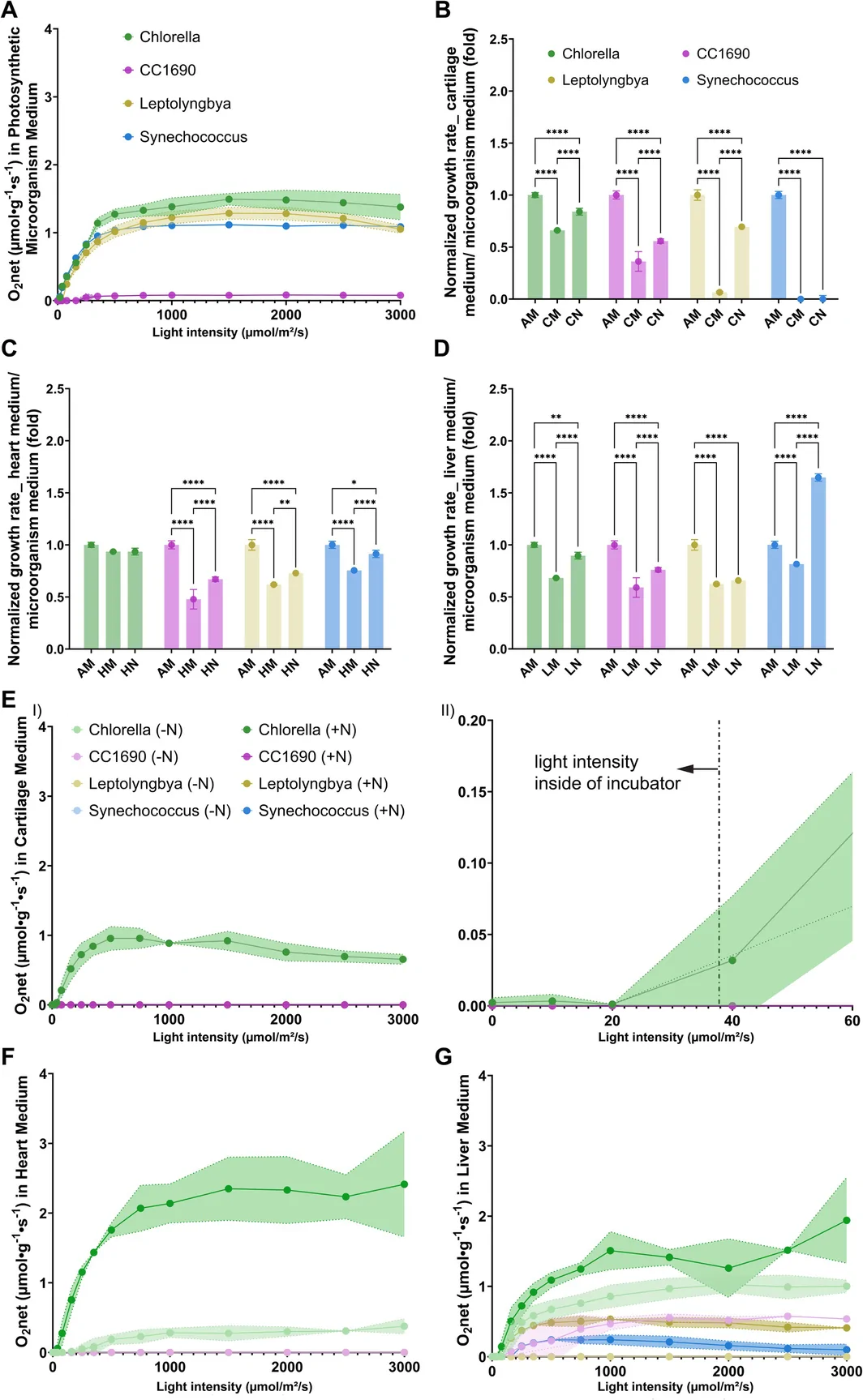
Growth and net oxygen production rate of photosynthetic microorganisms in different tissue culture media. (A) Net oxygen release profiles of the selected photosynthetic microorganisms in their respective microalgae or cyanobacteria culture media (AM) at 37°C, *n* = 2. Normalized growth rates of different photosynthetic microorganisms in CM and CM supplied with NaNO_3_ (CN) (B), HM and hear medium supplied with NaNO_3_ (HN) (C) and liver medium (LM) and liver medium supplied with NaNO_3_ (LN) (D), the growth rates are normalized by AM (*n* = 3). Two‐way ANOVA with Tukey's multiple comparisons test was applied to (B–D). *p ≤ 0.05, **p ≤ 0.01, ****p ≤ 0.0001. (E) Net specific oxygen release profiles of the photosynthetic microorganisms in medium of CM and CN under (I) light intensity ranges from 0 to 3000 μmol photons m^−2^ s^−1^ and (II) light intensity ranges from 0 to 60 μmol photons m^−2^ s^−1^. (F, G) Net oxygen release profiles of the photosynthetic microorganisms in HM and HN (F) and LM and LN (G) (*n* = 2).

When these four photosynthetic microorganisms were cultured in CN medium, a significant increase (*p* < 0.0001) in growth rate was observed compared to the control medium CM, as shown in Figure 4B. Specifically, the normalized average growth rate of *Chlorella*, *C. reinhardtii* CC 1690 and *Leptolyngbya* increased from about 0.66 to 0.84, 0.36 to 0.56 and 0.07 to 0.70, respectively, after adding nitrate. In contrast, *Synechococcus* demonstrated negative growth in both CM and CN. Given the previous observation in chlorophyll *a* content of *Synechococcus* within constructs over 28 days, we speculate that *Synechococcus* could survive but not grow in the CN medium. This is possible since cyanobacteria have evolved a range of adaptive strategies to ensure their survival under different stresses [69, 70].

Interestingly, *Synechococcus* exhibited growth in both HM (Figure 4C) and LM (Figure 4D), with a significantly increased growth rate (*p* < 0.0001) when additional nitrate was provided. Reflecting on the composition of the basic tissue media, CM lacks several key nutrients compared to LM and HM, such as vitamin B12 (cobalamin) and biotin (vitamin B7). This deficiency may partly explain the limited growth of *Synechococcus* in CM and CN. Previous studies have shown that among 306 tested microalgal species, about half required vitamin B12 and 5% required biotin [71]. However, one *Synechococcus* species has been reported to rely on both vitamin B12 and biotin for growth [72, 73, 74]. Notably, another *Synechococcus* species has demonstrated the ability to synthesize biotin [75, 76, 77] and some cyanobacteria, including *Synechococcus* sp. [78, 79], can produce pseudocobalamin, which serves as a substitute for cobalamin. This suggests that additional biotin and vitamin B12 may not always be critical for the growth of *Synechococcus*. It is also worth noting that photosynthetic microorganisms from different regions may have varying micronutrient demands [80]. Finally, other essential elements present in the B27 supplement (Table S6) used in LM and HM, but absent in CM, could further contribute to the superior growth conditions observed in those media.

Supplying NaNO_3_ not only stimulates the growth rate of all the photosynthetic microorganisms, but it also increases photosynthesis and does, therefore, result in increased oxygen release in the culture tissue media. For example, all the four photosynthetic microorganisms did not produce detectable oxygen in CM, whereas after supplying additional nitrate in CM, *Chlorella* showed positive oxygen release and the maximum net SOPR reached 0.957 ± 0.145 μmol O_2_ g^−1^ s^−1^ under 750 μmol photons m^−2^ s^−1^ of light intensity (Figure 4E, I), while under 37.8 μmol photons m^−2^ s^−1^ of incubator light intensity, the net oxygen release reached about 0.032 μmol O_2_ g^−1^ s^−1^ (Figure 4E, II) in 15 min (Table S4). It demonstrated our previous hypothesis that supplying additional N source is important for the growth and oxygen release of photosynthetic microorganisms.

The net oxygen release profile of photosynthetic microorganisms in CN also suggests that *Chlorella* was the only photosynthetic microorganism that could supply oxygen to ACPCs in the chondrogenesis constructs. Given that hypoxia promotes chondrogenesis [81, 82, 83], the negative formation of GAGs and Col II in the *Chlorella*‐based cartilage‐like constructs in Section 3.3 (Figure 3A,C) could be due to excessive oxygen supply. In the future, the bulk size and 3D structure of the constructs should be carefully considered when incorporating oxygen‐releasing photosynthetic microorganisms. Additionally, it is important to note that both energy production and matrix synthesis of cartilage‐derived cells can be inhibited if the oxygen concentration falls below 1% [84, 85]. Therefore, supplying suitable oxygen is crucial for engineered tissue constructs.

The positive effect of additional nitrate on oxygen production was also evident in both HM and LM. All four photosynthetic microorganisms could grow in both HM and HN media. However, only *Chlorella* exhibited oxygen production. The maximum net SOPR of *Chlorella* in HN and HM was 2.349 and 0.275 μmol O_2_ g^−1^ s^−1^, respectively, under a light intensity of 1500 μmol photons m^−2^ s^−1^ (Figure 4F). Notably, the maximum net SOPR of *Chlorella* in HN was even higher than in its own AM medium (1.493 μmol O_2_ g^−1^ s^−1^). This enhanced oxygen production rate in the HN may be attributed to the mixotrophic growth of *Chlorella*. Compared to photoautotrophic growth, mixotrophic conditions result in a higher algal respiration rate and greater photosynthetic oxygen evolution rate, with increased saturation light intensity and photon utilization efficiency [86]. Previous studies suggest that in the presence of organic carbon sources, the photochemical efficiency of Photosystem II (PSII) and overall photosynthesis efficiency are downregulated due to the suppression of the photosynthetic machinery [87]. However, this downregulation occurs only under nitrogen depletion conditions. Under nitrogen‐repletion conditions, the photosynthesis activity remains unaffected [86]. Interestingly, not only *Chlorella*, but also *C. reinhardtii* CC 1690, *Leptolyngbya* and *Synechococcus* demonstrated positive oxygen release in LM and/or LM + NaNO_3_ (LN). The observed maximum net SOPR for these three microorganisms was 0.576, 0.534 and 0.242 μmol O_2_ g^−1^ s^−1^, respectively (Figure 4G). This suggests that LM/LN is suitable for supporting the growth of various photosynthetic microorganisms and facilitating oxygen release for at least about a week. LM contains essential manganese trace elements, which are important for photosynthetic processes in PSII [88]. Additionally, zinc, copper and vanadium play critical roles in modulating photosynthetic electron transport, carbon fixation and nitrogen fixation within cyanobacteria and *C. reinhardtii* CC 1690 [89, 90, 91, 92]. Although, these results highlight the importance of supplying and optimizing trace elements and inorganic salt concentrations, particularly, those crucial for the structure and function of chlorophyll and PSII, within tissue culture media to support the successful growth and maintenance of photosynthetic microorganisms, a follow‐up study to validate this mechanism is suggested.

Further research should focus on optimizing culture conditions for distinct mammalian cells and photosynthetic microorganisms to improve their survival and functionality. Key areas to explore include evaluating various medium components, types of light, intensities and light/dark cycles. Additionally, it is critical to determine the optimal mixing ratios of photosynthetic microorganisms and mammalian cells. The application of individual or convergent biofabrication techniques will also be essential for effectively integrating mammalian cells with photosynthetic microorganisms. These findings may help clarify the dynamics of oxygen and nutrient exchange, enabling the development of tissue constructs that are specifically tailored to meet varying oxygen and nutrient demands. However, the successful development of co‐culture constructs comprising photosynthetic microorganisms and mammalian cells still poses some practical challenges. For example, their performance depends on optimized light delivery to prevent self‐shading [93], and the maintenance of compatible temperature ranges [94, 95]. For both in vitro and in vivo applications, careful selection of microorganism species is crucial to ensure biocompatibility and minimize the risk of immune responses [96, 97]. In this context, our study contributes by providing insights into several less commonly used photosynthetic microorganisms, thereby broadening the range of potential candidates. Scaling these 3D constructs towards clinically relevant sizes and physiologically characteristics introduces challenges, including limitations in macronutrient and trace element transport, as well as the need for long‐term mechanical stability. From a regulatory perspective, the clinical application of photosynthetic microorganisms requires rigorous safety assessments, well‐defined containment strategies and compliance with established guidelines for live biotherapeutic products [98, 99]. Addressing these requirements may help pave the way for advances in the field.

## 4 Conclusion

In summary, we evaluated the effects of using mammalian cells alongside various photosynthetic microorganisms in co‐culture conditions over a 28‐day culture period on metabolic activity and the functionality of engineered cartilage constructs. Although continuous exposure to white light for 28 days alters the function of mammalian cells, ACPCs outperformed MSCs. When photosynthetic microorganisms, such as *Chlorella*, *Leptolyngbya*, *Synechococcus* and *C. reinhardtii* CC 1690, were co‐cultured with ACPCs, we found that *Leptolyngbya* and *Synechococcus* did not adversely affect the chondrogenic capacity of ACPCs over the 28 days. In contrast, *C. reinhardtii* CC 1690 was unable to survive after 14 days and *Chlorella* suppressed chondrogenesis. Additionally, we found that the composition of different tissue culture media significantly influenced the growth and oxygen dynamics of photosynthetic microorganisms. All the investigated microorganisms released oxygen when cultured in trace elements‐enriched LM, highlighting the role of environmental factors in their photosynthetic activities. Notably, *Chlorella* was the only microorganism that released oxygen in cartilage culture medium, while *Leptolyngbya* and *Synechococcus* did not produce detectable oxygen under the same conditions. However, oxygen released by *Chlorella* affected the chondrogenesis of ACPCs, whereas ACPCs differentiated and produced cartilage‐like ECM when co‐cultured with *Leptolyngbya* and *Synechococcus*. These findings highlight the importance of selecting suitable photosynthetic microorganisms to support the growth of mammalian cells in large constructs when optimizing co‐culture conditions to supply oxygen to various tissue types. Additionally, it is crucial to maintain a balance between oxygen production and the specific nutritional needs of mammalian cells. Our study provides valuable insights for designing effective co‐culture systems and advancing tissue regeneration strategies that leverage photosynthetic microorganisms.

## Author Contributions

M.W., M.d.R. and J.M. conceived and designed the experiments. M.W., A.F.H., V.v.d.N and S.T.B. conducted the experiments. M.W., A.K.O., M.J., M.D.R. and J.M. analysed the data. M.W. wrote the original draft of the manuscript. All authors contributed to the revision of the manuscript. All authors read and approved the final manuscript.

## Funding

This research was supported by the SEED Fund of EWUU Alliance. Meng Wang thanks the China Scholarship Council (CSC202108440024) for financial support.

## Ethics Statement

No animal experiments were conducted.

## Conflicts of Interest

The authors declare no conflicts of interest.

## Supporting information


**Figure S1:** The comparison of measured OD750 as the observed growth parameter at 25°C and 37°C for *Chlamydomonas reinhardtii* CC1690 (*n* = 3).
**Table S1:** Composition of artificial seawater medium (for *Synechococcus* sp. and *Leptolyngbya* sp.), *Chlorella sorokiniana* medium and TAP medium (for *Chlamydomonas reinhardtii* CC1690).
**Figure S2:** Articular cartilage‐derived chondroprogenitor cells (ACPCs) remain metabolically active under continuous nitrogen exposure without continuous light illumination (*n* ≥ 12), one‐way ANOVA with Tukeys multiple comparisons test.
**Table S2:** Formula used for calculating photosynthetic microorganism cell number according to OD750 confirmed by Multiplate Reader (96‐well plate).
**Table S3:** Formula used for calculating photosynthetic microorganism dry biomass according to OD750 confirmed by 1 cm light path length cuvette.
**Table S4:** PFD plan used for specific oxygen production rate measurement.
**Table S5:** Concentration of essential nutrients that are important for microalgae growth in different culture media.
**Figure S3:** Chlorophy II *α* quantification of distinct photosynthetic microoganisms in the co‐cultured constructs after 28 days.
**Figure S4:** Metabolic activity of photosynthetic microorganisms with distinct microorganism densities in suspension culture (*n* = 3).
**Figure S5:** Immunohistochemistry staining of Collagen II in distinct constructs.
**Figure S6:** Immunohistochemistry staining of hypoxia factors, HiF‐1α and HBB, in distinct constructs (*n* = 3).
**Table S6:** Additives and supplements used in the tissue media.

## Data Availability

The data that support the findings of this study are available from the corresponding author upon reasonable request.
