# Supplementary material for: Co‐Culture of Mammalian Cells and Photosynthetic Microorganisms for Oxygen Supply in Engineered Tissues
Source: Cell Prolif. 2026 May 26:e70224. Online ahead of print. doi: 10.1111/cpr.70224 (PMC13325642; doi:10.1111/cpr.70224)
Supplement: Supplementary file 1 — Figure S1: The comparison of measured OD750 as the observed growth parameter at 25°C and 37°C for Chlamydomonas reinhardtii CC1690 (n = 3). Table S1: Composition of artificial seawater medium (for Synechococcus sp. and Leptolyngbya sp.), Chlorella sorokiniana medium and TAP medium (for Chlamydomonas reinhardtii CC1690). Figure S2: Articular cartilage‐derived chondroprogenitor cells (ACPCs) remain metabolically active under continuous nitrogen exposure without continuous light illumination (n ≥ 12), one‐way ANOVA with Tukeys multiple comparisons test. Table S2: Formula used for calculating photosynthetic microorganism cell number according to OD750 confirmed by Multiplate Reader (96‐well plate). Table S3: Formula used for calculating photosynthetic microorganism dry biomass according to OD750 confirmed by 1 cm light path length cuvette. Table S4: PFD plan used for specific oxygen production rate measurement. Table S5: Concentration of essential nutrients that are important for microalgae growth in different culture media. Figure S3: Chlorophy II α quantification of distinct photosynthetic microoganisms in the co‐cultured constructs after 28 days. Figure S4: Metabolic activity of photosynthetic microorganisms with distinct microorganism densities in suspension culture (n = 3). Figure S5: Immunohistochemistry staining of Collagen II in distinct constructs. Figure S6: Immunohistochemistry staining of hypoxia factors, HiF‐1α and HBB, in distinct constructs (n = 3). Table S6: Additives and supplements used in the tissue media. [file CPR-9999-e70224-s001.docx]

# Co-Culture of Mammalian Cells and Photosynthetic Microorganisms for Oxygen Supply in Engineered Tissues

#

*Meng Wang**^1,2^, Ahmad Furqan Hala^3^, Vera van der Niet^3^, Sebastian T. Bok*^1,2^*,* *Aylin Kara Özenler^1,2^, Marcel Janssen^3^, Maria J. Barbosa^3^, Dirk Martens^3^, Rene H Wijffels^3^, Jos Malda ^1,2,4 #^, Mylène de Ruijter ^1,2,4^*

*^1^* Department of Orthopaedics, University Medical Center Utrecht, Utrecht University, Utrecht, Heidelberglaan 100, 3584 CX, The Netherlands

^2^ Regenerative Medicine Center Utrecht, Uppsalalaan 8, 3584 CT, Utrecht, The Netherlands

^3^Wageningen University, Bioprocess Engineering, AlgaePARC, P.O. Box 16, 6700 AA,

Wageningen, Netherlands

^4^ Department of Clinical Sciences, Faculty of Veterinary Medicine, Utrecht University, Yalelaan 1, 3584 CL, Utrecht, The Netherlands

*^#^* Corresponding author: [j.malda@umcutrecht.nl](mailto:j.malda@umcutrecht.nl)

## Supplemental materials


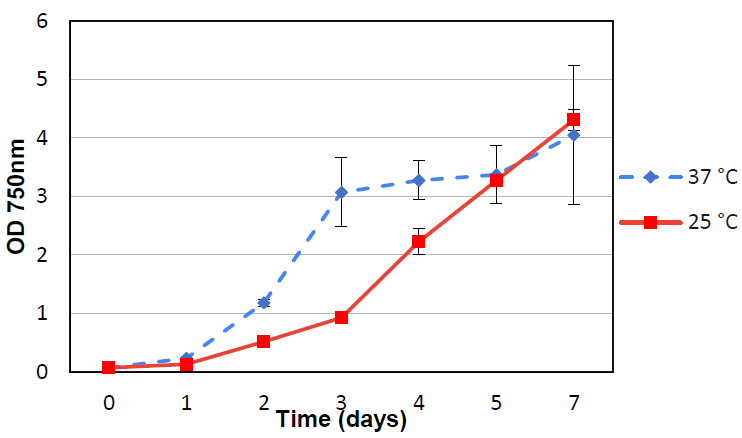


Supplemental Figure 1. The comparison of measured OD750 as the observed growth parameter at 25°C and 37°C for *C. reinhardtii* CC1690 (n = 3).

Supplemental Table 1. Composition of artificial seawater medium (for *Synechococcus* sp. and *Leptolyngbya* sp.), *Chlorella sorokiniana* medium and TAP medium (for *Chlamydomonas reinhardtii* CC1690).

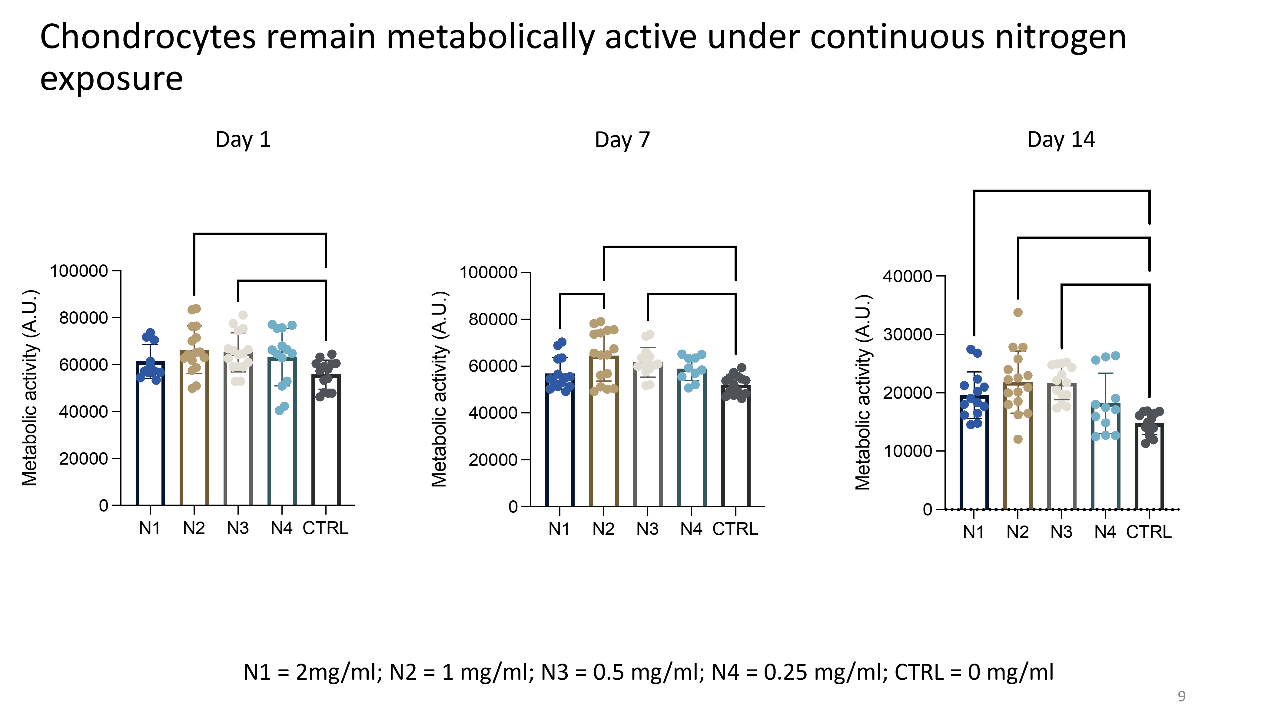


Supplemental Figure 2. Articular cartilage-derived chondroprogenitor cells (ACPCs) remain metabolically active under continuous nitrogen exposure without continuous light illumination­­ (n ≥ 12), One-way ANOVA with Tukeys multiple comparisons test. *P ≤ 0.05. N1=2 g∙L^-1^, N2=1 g∙L^-1^, N3=0.5 g∙L^-1^, N4=0.25 g∙L^-1^, CTRL=0 g∙L^-1^.

Supplemental Table 2. Formula used for calculating photosynthetic microorganism cell number according to OD750 confirmed by Multiplate Reader (96-well plate). OD should below 0.1. DC: dilute coefficient.

|  | Microalgal species | Cell counting (*10^6^ cells ml^-1^) | R^2^ |
| --- | --- | --- | --- |
| 96-well plate | *Chlorella sorokiniana* | 18.181*OD750*2.9*DC + 58.552 | 0.9182 |
|  | *Chlamydomonas reinhardtii* CC1690 | 4.008*OD750*1.29*DC + 1.407 | 0.953 |
|  | *Leptolyngbya* sp. | (27.421*OD750*2.21*DC*100 - 59.365)/100 | 0.8713 |
|  | *Synechococcus* sp. | 107.020*OD750*2.74*DC + 8.049 | 0.9673 |

Supplemental Table 3. Formula used for calculating photosynthetic microorganism dry biomass according to OD750 confirmed by 1 cm light path length cuvette. OD should below 0.3.

|  | Microalgal species | Cell counting (*10^6^ cells ml^-1^) | R^2^ |
| --- | --- | --- | --- |
| 96-well plate | *Chlorella sorokiniana* | 0.3354*OD750 - 0.3868 | 0.9367 |
|  | *Chlamydomonas reinhardtii* CC1690 | 0.657*OD750 + 0.031 | 0.99 |
|  | *Leptolyngbya* sp. | 0.4672*OD750 - 0.2422 | 0.9565 |
|  | *Synechococcus* sp. | 0.334*OD750 + 0.2087 | 0.9132 |

Supplemental Table 4. PFD Plan used for specific oxygen production rate measurement.


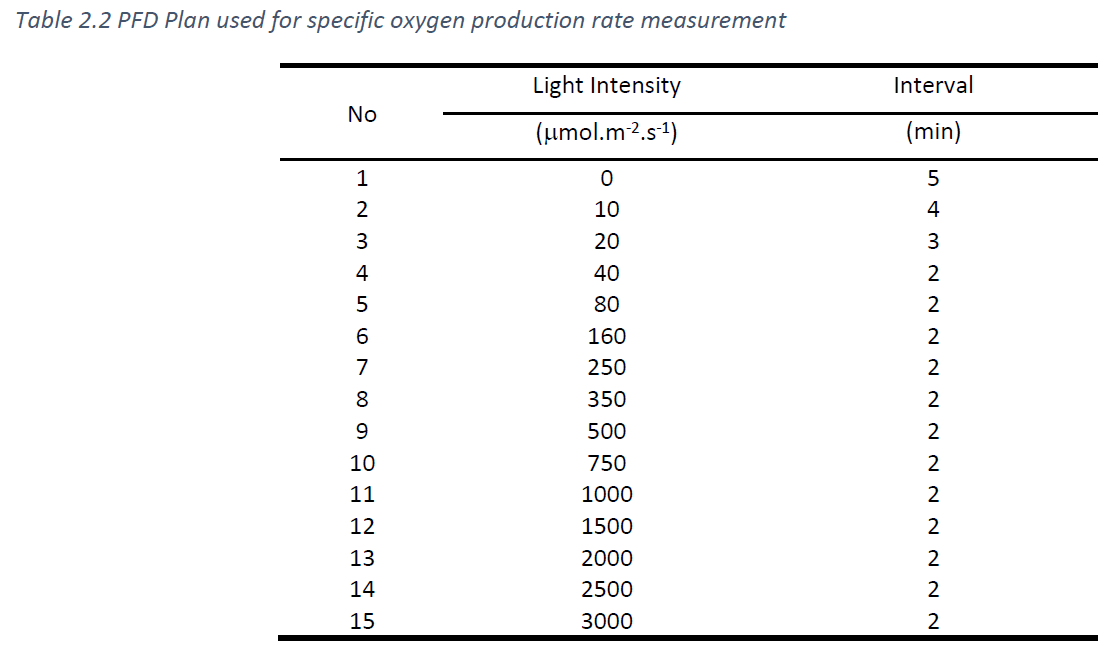


Supplemental Table 5. Concentration of essential nutrients that are important for microalgae growth in different culture media.

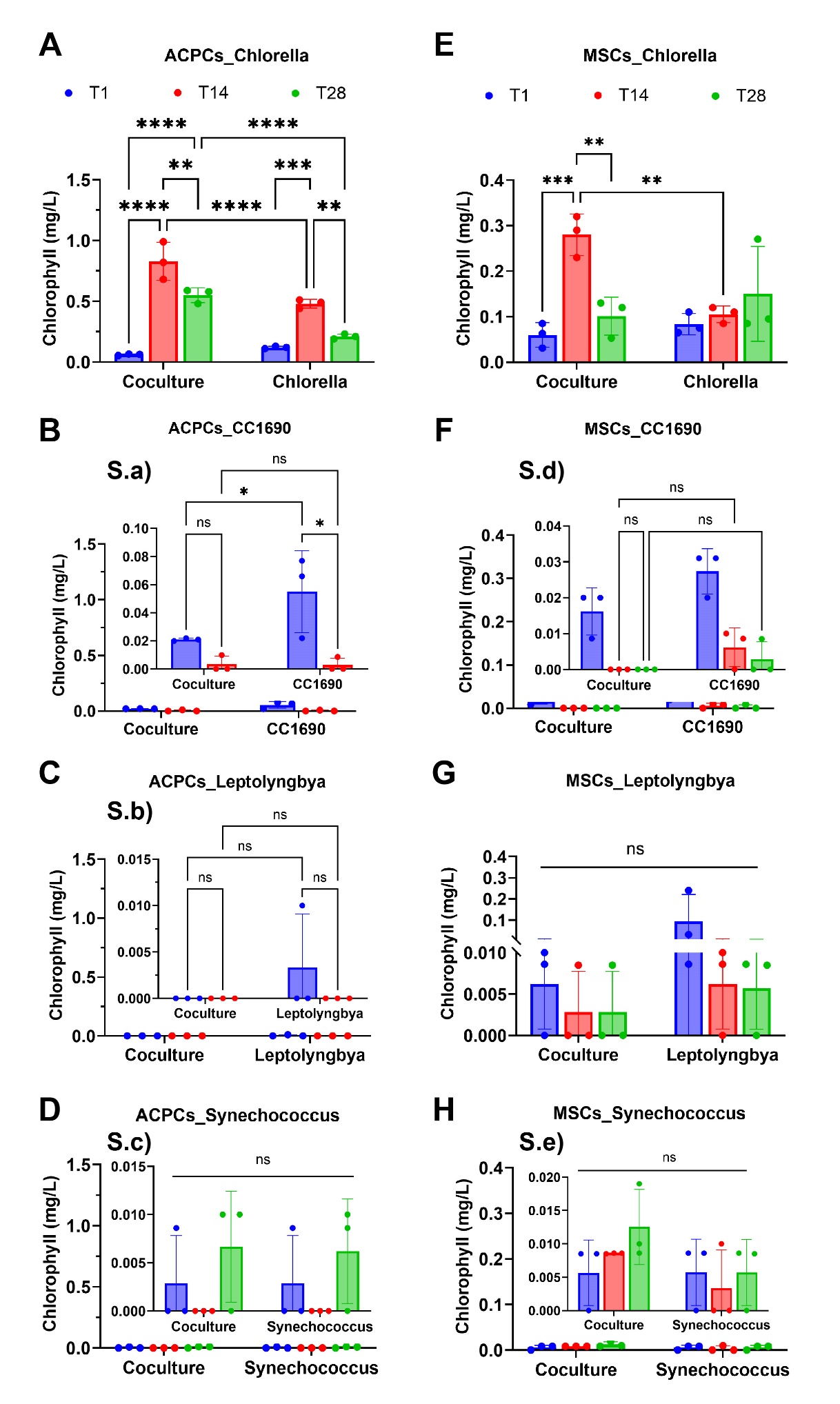


Supplemental Figure 3. Chlorophy II *α* quantification of distinct photosynthetic microoganisms in the co-cultured constructs after 28 days. Chlorophy II *α* content in articular cartilage-derived chondroprogenitor cells (ACPCs) co-cultured constructs incorporated with *Chlorella* (A), *C. reinhardtii* CC 1690 (B), *Leptolyngbya* (C), and *Synechococcus* (D). Chlorophy II *α* content in bone marrow-derived mesenchymal stromal cells (MSCs) co-cultured constructs incorporated with *Chlorella* (E), *C. reinhardtii* CC 1690 (F), *Leptolyngbya* (G), and *Synechococcus* (H). n = 3. Two-way ANOVA with Šídák's multiple comparisons test for panel A, D, and E, Two-way ANOVA with Uncorrected Fisher's LSD for panel B and C, Two-way ANOVA with Tukeys multiple comparisons test for panel F, G and H. *P ≤ 0.05, **P ≤ 0.01, ***P ≤ 0.001 and ****P ≤ 0.0001.


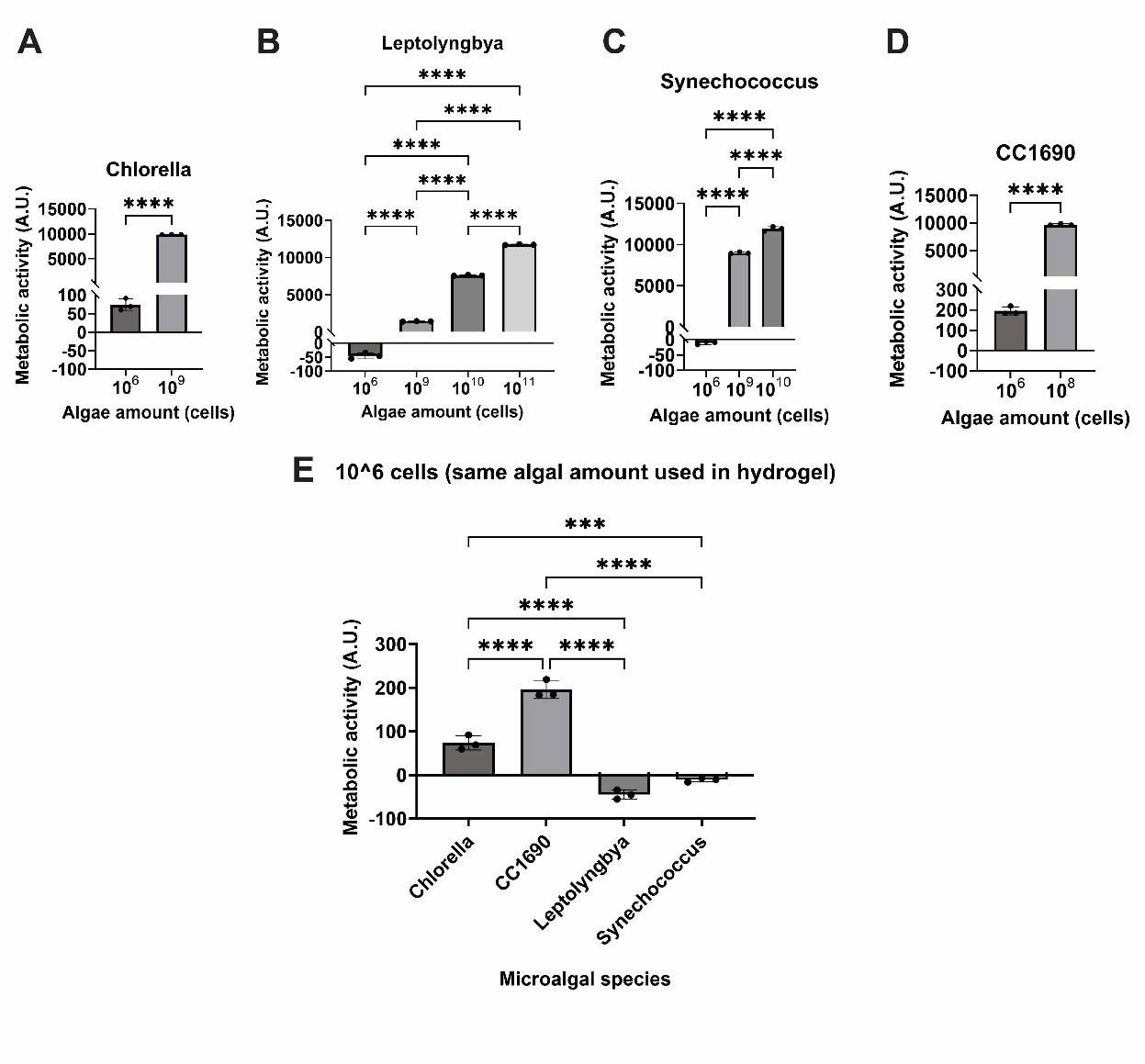


Supplemental Figure 4. Metabolic activity of photosynthetic microorganisms with distinct microorganism densities in suspension culture (n=3). (A) *Chlorella*, Unpaired t test with Welch's correction. (B) *C. reinhardtii* CC 1690, Unpaired t test with Welch's correction. (C) *Leptolyngbya*, Ordinary one-way ANOVA with Tukey's multiple comparisons test. (D) *Synechococcus,* Ordinary one-way ANOVA with Tukey's multiple comparisons test. (E) Distinct photosynthetic microorganisms with 10^6^ cells/ml of cell density, Ordinary one-way ANOVA with Tukey's multiple comparisons test. ***P ≤ 0.001, ****P ≤ 0.0001. Metabolic activity of *Leptolyngbya* sp. and *Synechococcus* sp. can be detected when increasing microalgal cell number.


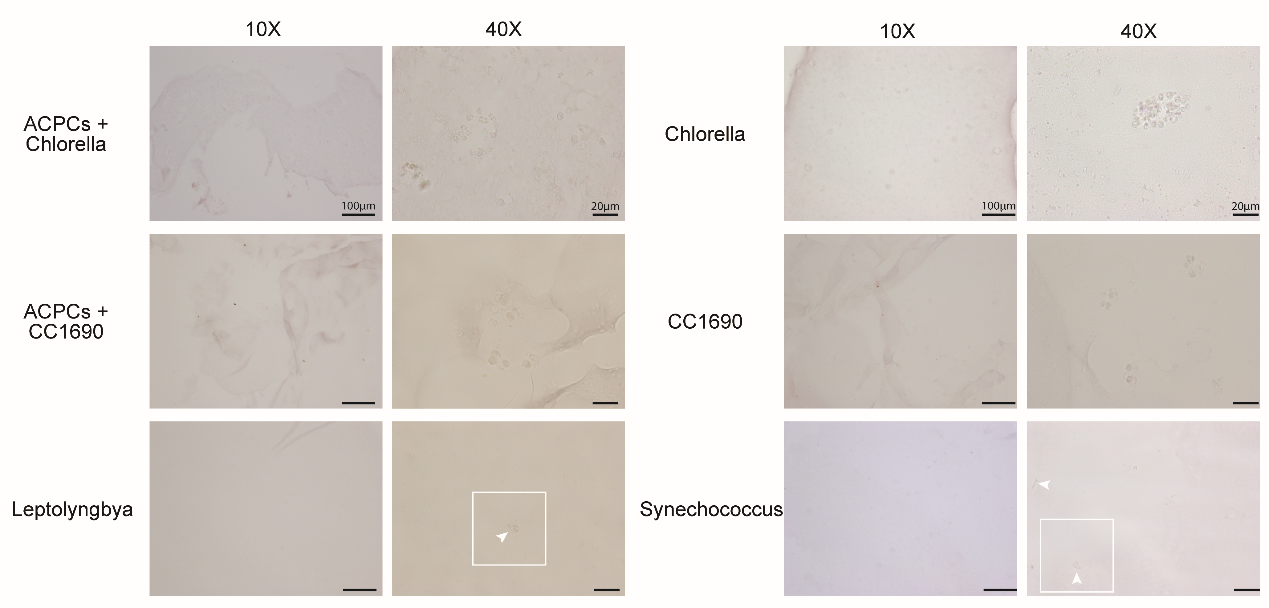


Supplemental Figure 5. Immunohistochemistry staining of Collagen II in distinct constructs. Scale bar: 10X is 100 µm, 40X is 20 µm. The white arrows indicate *Leptolyngbya* or *Synechococcus* inside of constructs. n = 3.

_
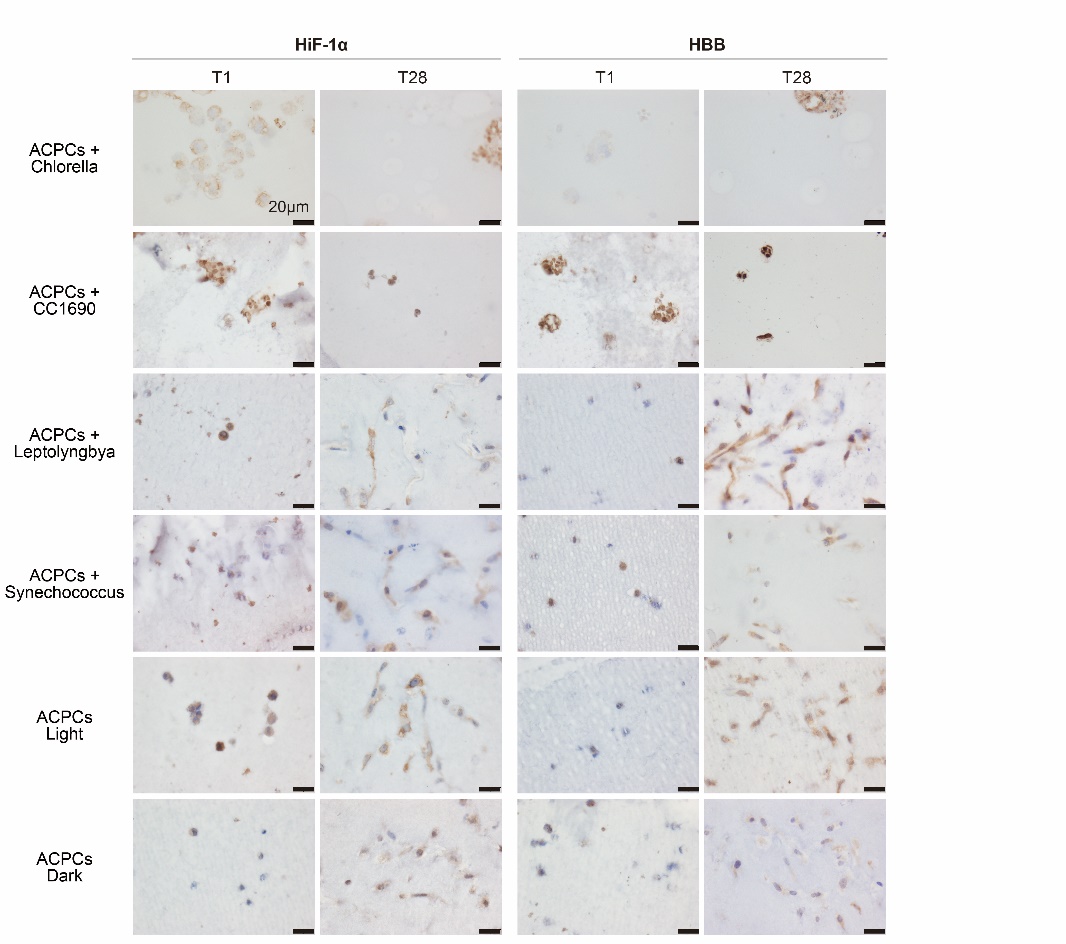
_

Supplemental Figure 6. Immunohistochemistry staining of hypoxia factors, HiF-1α and HBB, in distinct constructs (n = 3). Scale bar: 20 µm.

Supplemental Table 6. Additives and supplements used in the tissue media. p.s., Recombinant human transforming growth factor was not added to chondrogenic medium when measuring both growth rate and net oxygen production rate by photosynthetic microorganism in Figure 4.
